# Inhibition of breast cancer cell invasion by melatonin is mediated through regulation of the p38 mitogen-activated protein kinase signaling pathway

**DOI:** 10.1186/bcr2794

**Published:** 2010-12-17

**Authors:** Lulu Mao, Lin Yuan, Lauren M Slakey, Frank E Jones, Matthew E Burow, Steven M Hill

**Affiliations:** 1 Department of Structural and Cellular Biology, Tulane University School of Medicine, 1430 Tulane Avenue, New Orleans, LA 70112, USA; 2 Tulane Cancer Center, Tulane University School of Medicine, 1430 Tulane Avenue, New Orleans, LA 70112, USA; 3 Department of Cell and Molecular Biology, Tulane University, 2000 Percival Stern, New Orleans, LA 70118, USA; 4 Department of Medicine, Tulane University School of Medicine, 1430 Tulane Avenue, New Orleans, LA 70112, USA

## Abstract

**Introduction:**

The pineal gland hormone, melatonin, has been shown by numerous studies to inhibit the proliferation of estrogen receptor α (ERα)-positive breast cancer cell lines. Here, we investigated the role of melatonin in the regulation of breast cancer cell invasion.

**Methods:**

Three invasive MCF-7 breast cancer cell clones - MCF-7/6, MCF-7/Her2.1, and MCF-7/CXCR4 cells - were employed in these studies. All three cell lines exhibited elevated phosphorylation of the ERK1/2 and p38 mitogen-activated protein kinase (MAPK) as determined by Western blot analysis. The effect of melatonin on the invasive potential of these human breast cancer cells was examined by matrigel invasion chamber assays. The expression and proteinase activity of two matrix metalloproteinases (MMPs), MMP-2 and MMP-9, were analyzed by Western blot analysis and gelatin zymography, respectively.

**Results:**

Melatonin (10^-9 ^M) significantly suppressed the invasive potential of MCF-7/6 and MCF-7/Her2.1 cells as measured by matrigel invasion chamber assays, and significantly repressed the proteinase activity of MMP-2 and MMP-9. In MCF-7/CXCR4 cells, melatonin significantly inhibited stromal-derived factor-1 (SDF-1/CXCL12) induced cell invasion and activity of MMP-9. Elevated expression of the MT1 melatonin receptor further enhanced, while luzindole, an MT1/MT2 antagonist, abrogated melatonin's anti-invasive effect, suggesting that melatonin's effect on invasion is mediated, principally, through the MT1 receptor. Furthermore, melatonin repressed the phosphorylation of p38 MAPK in MCF-7/Her2.1 cells and blocked stromal-derived factor-1 (SDF-1) induced p38 phosphorylation in MCF-7/CXCR4 cells. SB230580, a p38 inhibitor, was able to mimic, while transfection of the cells with a constitutively-active MKK6b construct blocked melatonin's effect on cell invasion, suggesting that the anti-invasive action of melatonin is mediated through the p38 pathway.

**Conclusions:**

Melatonin exerts an inhibitory effect on breast cancer cell invasion through down-regulation of the p38 pathway, and inhibition of MMP-2 and MMP-9 expression and activity.

## Introduction

Over the last several decades, melatonin's growth-inhibitory action in breast cancer has been studied extensively both *in vivo *and *in vitro*. In contrast, only a minimal amount of work has been done with regard to the role of melatonin in breast cancer invasion and metastasis. It has been observed in several early correlative studies that the plasma level of melatonin is significantly reduced in cancer patients with metastatic disease as compared with those without metastases [1,2]. In 1998, Cos and colleagues [3] reported that physiological concentrations of melatonin (10^-9 ^M) significantly reduced the invasive capacity of MCF-7 human breast cancer cells as measured by Falcon invasion chamber assays, a modified Boyden chamber assay, and that melatonin could enhance the expression of the adhesion proteins, E-cadherin and β_1 _integrin. In addition, melatonin administration has been shown to reduce the incidence of metastases in several *in vivo *studies [4-6].

Collectively, these results suggest that melatonin may exert an inhibitory influence on breast cancer cell invasion and metastasis, possibly by decreasing cell attachment to the basement membrane. However, there has been no further exploration of melatonin's anti-invasive action and mechanism(s) since the work by Cos and colleagues in 1998. A major obstacle to a better understanding of melatonin's role in breast cancer invasion and metastasis is the lack of a cell line that exhibits a strong invasive potential but that is also estrogen receptor-alpha (ERα)-positive and melatonin-responsive. The ERα-positive MCF-7 cell, which has been well characterized and extensively used in the *in vitro *studies examining melatonin's anti-proliferative effect and which has been shown to be responsive to melatonin-mediated growth inhibition, is widely regarded as poorly invasive. Thus, the standard MCF-7 breast tumor cell line is not a good model for invasion/metastasis studies. Unfortunately, the highly invasive ERα-negative MDA-MB-231 cells are unresponsive to melatonin's growth-inhibitory actions and thus are not a reasonable model to study melatonin's actions on invasion. Therefore, an alternative cell line that exhibits high invasive potential but that still retains the melatonin responsiveness is essential for a model system in which to study melatonin's actions on breast cancer invasion.

Here, we have used three invasive breast cancer cell lines. The MCF-7/6 cells were derived from parental MCF-7 cells by selection for metastatic potential by serial passaging in nude mice [7]. As compared with the MCF-7/AZ line (a parental MCF-7 cell clone renamed by the group of Marc Mareel, Gent University Hospital, Gent, Belgium), MCF-7/6 cells are invasive in the chick heart embryo invasion assay [7] and spontaneously metastasize in nude mice after subcutaneous injection [8]. These cells have been demonstrated to be ERα-positive and progesterone receptor (PR)-positive.

A second cell line used in our studies is the MCF-7/Her2.1 cell line, which has been stably transfected with and overexpresses the wild-type human Her2/neu (C-erbB2) receptor. According to previous studies, receptor tyrosine kinase Her2/neu plays an important role in the malignant progression of breast cancer [9]. Amplification and overexpression of Her2/neu occur in approximately 15% to 30% of primary breast tumors and correlate with the nodal metastases and poor prognosis [10]. It was previously shown that Her2/neu overexpression in breast cancer cells frequently leads to hyper-activation of mitogen-activated protein kinase (MAPK) signaling pathways [11]. Moreover, overexpression of Her2/neu not only induces the invasive capacity in mammary epithelial cells but also promotes the invasiveness of breast cancer cells *in vitro *[12,13] and induces metastasis in animal models [13,14]. Although the effect of Her2/neu on metastasis is well documented, the mechanism underlying the effect of Her2/neu on breast cancer invasion and metastasis is not fully understood. A recent study by Ke and colleagues [15] showed that, in human mammary epithelial cells, expression and activity of matrix metalloproteinase (MMP)-2 and MMP-9, two members of the MMP family which play an important role in the degradation of the extracellular matrix, were upregulated in response to overexpression of Her2/neu and that the regulation of MMP-2 and MMP-9 by Her2/neu may be mediated through the p38 MAPK and PI3K (phosphoinositide 3-kinase) signaling pathways. These results suggest that the effects of Her2/neu on breast cancer cell invasion may impinge on target molecules to orchestrate the degradation of the extracellular matrix through simultaneous activation of multiple signaling pathways.

Also used in our studies is an MCF-7 clone (MCF-7/CXCR4) that overexpresses the chemokine receptor CXCR4, a recently described key regulator of breast cancer invasion and metastasis [16]. Through interaction with its cognate ligand, the chemokine stromal-derived factor-1 (SDF-1/CXCL12), CXCR4 is proposed to direct homing of breast cancer cells to particular sites of metastases [16]. It has been demonstrated that downregulation of CXCR4 inhibits *in vitro *invasiveness of breast cancer cell and blocks breast cancer metastasis *in vivo *[17,18]. Several lines of evidence suggest that the effect of the SDF-1/CXCR4 axis on cell invasion may involve activation of multiple signaling pathways, including the p38 MAPK pathway [19-21].

Although MCF-7/6, MCF-7/Her2.1, and MCF-7/CXCR4 breast cancer cells acquire their malignant phenotypes through different mechanisms and approaches, their invasive potential may be enhanced through activation of a common intracellular signaling pathway that plays an essential role in regulating cancer cell invasion and metastasis. According to previous studies [22-25], the p38 MAPK is a central kinase in a common pathway that plays an important role in breast cancer invasion and metastasis by modulating the expression and activity of molecules involved in the degradation of extracellular matrix (that is, MMP-2 and MMP-9). Additionally, p38 MAPK has been reported to be activated by both the Her2/neu and SDF-1/CXCR4 pathways [15,19-21]. Moreover, p38 MAPK activity has been shown to be regulated by cAMP [26-30]. Considering the above results and the fact that melatonin, via its MT1 receptor, regulates the intracellular concentration of cAMP in breast cancer cells [31], we hypothesize that melatonin plays an inhibitory role in breast cancer cell invasion by modulating the activation of the p38 MAPK pathway. Our results demonstrated that melatonin suppresses the *in vitro *invasive potential of breast cancer cells by altering the phosphorylation of p38 MAPK and the downstream activity of MMP-2 and MMP-9.

## Materials and methods

### Chemicals and reagents

All chemicals and tissue culture reagents were purchased from Sigma-Aldrich (St. Louis, MO, USA). Cell culture medium, Dulbecco's modified eagle medium (DMEM)/F-12 (1:1), RPMI 1640, and fetal bovine serum (FBS) were purchased from Gibco BRL (now part of Invitrogen Corporation, Carlsbad, CA, USA). The FuGENE 6 transfection reagent was purchased from Roche (Indianapolis, IN, USA). SDF-1/CXCL12 was purchased from Research Diagnostics Inc. (now part of Fitzgerald Industries International, Acton, MA, USA).

### Cell lines and cell culture

Eight human breast cancer cell lines were used in these studies. The MDA-MB-231 human breast cancer cell was purchased from American Type Culture Collection (Manassas, VA, USA). The parental MCF-7 cell was obtained from the laboratory of the late William L McGurie (San Antonio, TX, USA). The MCF-7/6 and MCF-7/AZ (control MCF-7 cell for MCF-7/6) cells were kindly provided by Marc Mareel. The MCF-7/Her2.1 (stably transfected with Her2/neu in pcDNA3.1 vector and overexpresses wild-type Her2/neu) and its control cell MCF-7/vec were generously provided by Frank E Jones (Tulane University, New Orleans, LA, USA). The MCF-7/CXCR4 cell line (stably transfected with CXCR4 in pcDNA3 vector and overexpresses CXCR4) and its control counterpart MCF-7/pcDNA3 were gifts from Matthew E Burow (Tulane University, New Orleans, LA, USA). All cell lines, except MCF-7/6 and MCF-7/AZ, were cultured in RPMI 1640 medium supplemented with 10% FBS (Gibco BRL), 50 mM minimum essential medium non-essential amino acids, 1 mM sodium pyruvate, 2 mM glutamine, 10 mM basal medium eagle, 100 mg/mL streptomycin, and 100 U/mL penicillin. The MCF-7/6 and MCF-7/AZ cells were cultured in DMEM/F-12 (1:1) with the same supplements described above. These cell lines were routinely maintained at 37°C in a humidified atmosphere of 5% CO_2 _and 95% air.

### Total RNA extraction and reverse transcriptase-polymerase chain reaction analysis

Reverse transcriptase-polymerase chain reaction (RT-PCR) analysis was performed to confirm the overexpression of MT1 in MT1-transfected cells. Total cellular RNA was isolated using the TRIzol reagent (Invitrogen Corporation) in accordance with the manufacturer's instructions. Reverse transcription was performed on 1 μg of total RNA using Superscript II RNase H- reverse transcriptase (Invitrogen Corporation) and 200 ng random hexadeoxynulceotide primers in 20-μL reaction volumes containing 3 mM MgCl_2_, 10 mM DTT, 75 mM KCl, and 0.5 mM dNTP. The polymerase chain reaction (PCR) amplification was conducted using primer sets as previously described [32]. Aliquots of the PCR products were separated on a 1% agarose gel.

### Real-time reverse transcriptase-polymerase chain reaction analysis

Real-time RT-PCR was performed to determine the mRNA levels of MMP-9 in MCF-7 cells transiently transfected with CAMKK6b. Total cellular RNA was isolated using the PerfectPure RNA Cultured Cell Kit (5 Prime, Gaithersburg, MD, USA) in accordance with the manufacturer's instructions. Reverse transcription was performed on 1 μg of total RNA using Superscript II RNase H- reverse transcriptase (Invitrogen Corporation) and 200 ng random hexadeoxynulceotide primers in 20-μL reaction volumes containing 3 mM MgCl_2_, 10 mM DTT, 75 mM KCl, and 0.5 mM dNTP. Real-time PCR was carried out in 20 μL of PCR mixture containing 10 μL of 2× iQ SYBR Green Supermix and 1 μL of each cDNA sample on an iCycler iQ real-time detection system (Bio-Rad Laboratories, Inc., Hercules, CA, USA) in triplicates and recorded in real time and analyzed using the accompanying program (iCycler iQ real-time PCR detection system software, version 3.0A; Bio-Rad Laboratories, Inc.). The level of the 18s ribosomal RNA was also determined by real-time RT-PCR in each cDNA sample to normalize the expression of MMP-9. The primers used were as follows: human MMP-9: forward primer, 5'-TGACAGCGACAAGAAGTG-3', and reverse primer, 5'-CAGTGAAGCGGTACATAGG-3'; 18s ribosomal RNA: forward primer, 5'-TTGACGGAAGGGCACCACCAG-3', and reverse primer, 5'-GCACCACCACCCACGGAATCG-3'. Melt curve analysis was performed at the end of each PCR to confirm the specificity of the PCR product. Threshold cycle (Ct) values of MMP-9 among samples were compared after correction for 18s expression. The ratio of MMP-9 versus the corresponding 18s of each sample was determined on the basis of the equation MMP-9/18s = 2^Ct(18s) - Ct(MMP-9)^. The ratio of MMP-9/18s was compared among samples, and the fold change of MMP-9 expression was obtained by setting the values from vector-transfected cells to 1.

### Cell proliferation assay

For melatonin response studies, MCF-7, MCF-7/AZ, MCF-7/6 cells, MCF-7/vec, MCF-7/Her2.1, and MDA-MB-231 cells were seeded at a density of 2.0 × 10^4 ^cells per well, serum-starved for 24 hours, and treated with melatonin (10^-9 ^M), or diluent (0.00001% ethanol), in complete medium supplemented with 10% FBS. Cells were counted after 6 days of melatonin exposure on a hemacytometer using trypan blue to select for viable cells.

### Protein extraction and Western blot analysis

Cells were harvested and then lysed in a protein extraction buffer containing Tris (50 mM, pH 7.4), EDTA (20 mM), NP-40 (0.5%), NaCl (150 mM), phenylmethylsulfonyl fluoride (0.3 mM), NaF (1 mM), NaVO_4 _(1 mM), dithiothreitol (1 mM), aprotinin (1 μg/mL), leupeptine (1 μg/mL), and pepstatin (1 μg/mL). The cell lysates were centrifuged for 10 minutes at 10,000 *g *at 4°C. Protein concentrations of the supernatants were determined using a protein assay kit (Bio-Rad Laboratories, Inc.). Total protein (50 μg per sample) was electrophoretically separated on a 10% SDS-polyacrylamide gel and electroblotted onto a Hybond membrane. After incubation with 5% non-fat milk in Tris-buffered saline containing 0.1% Tween, the immunoblots were probed with antibodies to MT1, Her2/neu (Santa Cruz Biotechnology, Inc., Santa Cruz, CA, USA), CXCR4 (Abcam, Cambridge, MA, USA), phospho-p38 (Thr180/Tyr182), phospho-p44/42 MAPK (Thr202/Tyr204) (Cell Signaling Technology, Inc., Danvers, MA, USA), MKK6 (Millipore Corporation, Billerica, MA, USA), or phospho-ETS1 (Thr38) (Invitrogen Corporation). The same blots were stripped and reprobed with antibodies to β-actin (Sigma-Aldrich), p38, p44/42 MAPK (Cell Signaling Technology, Inc.), GAPDH (glyceraldehyde-3-phosphate dehydrogenase), or ETS1 (Millipore Corporation), respectively. For MMP-2 and MMP-9 expression studies, the enriched conditioned medium from each treatment group was electrophoretically separated on a 10% SDS-polyacrylamide gel, and the blots were probed with anti-MMP-2 and anti-MMP-9 antibodies (Chemicon, now part of Millipore Corporation).

### Transient transfection

MCF-7/6 or MCF-7/Her2.1 cells were seeded in 150-mm^2 ^cell culture flasks at a density of 3.16 × 10^6 ^cells per flask in DMEM/F-12 (1:1) medium or RPMI-1640 supplemented with 10% FBS. After 24-hour serum starvation, cells were then transfected with 7.9 μg of pcDNA3.1-MT1 or pcDNA3.1 empty vector or with 7.9 μg of CA-MKK6b (a dominant-positive MKK6b construct provided by Matthew E Burow) or pcDNA3 empty vector, using the FuGENE 6 transfection reagent. Twenty-four hours following transfection, these cells were seeded for matrigel invasion assays.

### Matrigel invasion chamber assay

The invasive potential of breast cancer cells was assessed *in vitro *in matrigel-coated invasion Chambers (BD BioCoat Matrigel Invasion Chamber; Becton Dickinson Biosciences, Franklin Lakes, NJ, USA) in accordance with the manufacturer's instructions. Briefly, cells in log phase of growth were serum-starved for 24 hours prior to seeding, detached by brief trypsinization, and resuspended in medium containing the appropriate treatment. The matrigel invasion inserts were rehydrated and prepared as described in the manufacturer's instructions. Cells (5 × 10^4 ^cells/mL in 0.5 mL serum-free medium) were added in suspension to the upper chamber, and medium (0.75 mL, supplemented with 10% FBS as chemoattractant) containing the same treatment was added to the bottom well. After incubation for 4 or 6 days, the non-invasive cells were removed from the upper surface of the membrane, and the invasive cells on the under surface of the membrane were stained with a Diff-Quick staining kit (Dade Behring, now part of Siemens Healthcare Diagnostics, Deerfield, IL, USA) and counted microscopically at 100× magnification. Five fields per membrane were randomly selected and counted in each group. The percentage of invasive cells was calculated as the percentage invasion through the matrigel membrane relative to the migration through the control membrane, as described in the manufacturer's instructions. Because Cos and colleagues [3] reported that treatment of MCF-7 cells with melatonin for 6 days reduced cell invasion, we first confirmed their results by incubating the cells for 4 or 6 days (as described above) when examining melatonin's effect on the invasion of MCF-7/6 and MCF-7/Her2.1 cells. These results were confirmed in the more common 2-day invasion assays (data not shown). In these assays, the underside of the membrane was pre-coated with fibronectin (20 ng/mL, as chemoattractant) for 1 hour at 37°C after rehydration, and the cells were seeded at a higher density (2 to 3 × 10^6 ^cells/mL) and incubated for 2 days. As similar results were obtained using these different time frames, we chose to employ the 2-day invasion assays in our subsequent experiments.

### Preparation of conditioned medium

MCF-7/6 or MCF-7/CXCR4 cells were cultured in DMEM/F-12 (1:1) or RPMI-1640 (respectively) supplemented with 10% FBS until confluency. The cells were washed with phosphate-buffered saline three times and then incubated in serum-free medium containing the appropriate treatments. The conditioned medium was collected, and an equal amount of the medium (0.5 mL) was concentrated approximately 10-fold by centrifugation at 14,000 *g *using Microcon YM-30 columns (Millipore Corporation). The volume of the concentrated medium was measured, and a normalized volume of it (15 μL) was stored at -70°C until used for Western blot analysis or gelatin zymography.

### Gelatin zymography

The activity of MMP-2 and MMP-9 in the conditioned medium was determined by gelatin zymography. Briefly, the conditioned medium (15 μL per sample) was mixed with an equal volume of 2 × SDS sample buffer (Invitrogen Corporation) and subjected under non-reducing conditions to SDS-polyacrylamide gel polymerized with 1 mg/mL gelatin. Following electrophoresis, the gels were incubated in a renaturing buffer (2.5% Triton X-100) with gentle agitation to remove SDS and then incubated in a developing buffer (50 mM Tris-HCl buffer, pH 7.4, and 10 mM CaCl_2_) overnight at 37°C. Gels were then stained with SimplyBlue SafeStain (Invitrogen Corporation) and destained in the Gel-Drying solution (Invitrogen Corporation). Gelatinase activity was visualized as clear bands against the blue-stained background. Molecular sizes were determined from mobility using gelatin zymography standards (Chemicon).

### Statistical analysis

Data are represented as the mean ± the standard error of the mean. The statistical significance at 95% confidence level was determined by one-way analysis of variance followed by a Student Newman-Keuls *post hoc *test analysis using the Statview software (SAS Institute, Inc., Cary, NC, USA).

## Results

### Characterization of MT1 expression and melatonin responsiveness of a panel of human breast cancer cells with various invasive potentials

**Figure 1:**
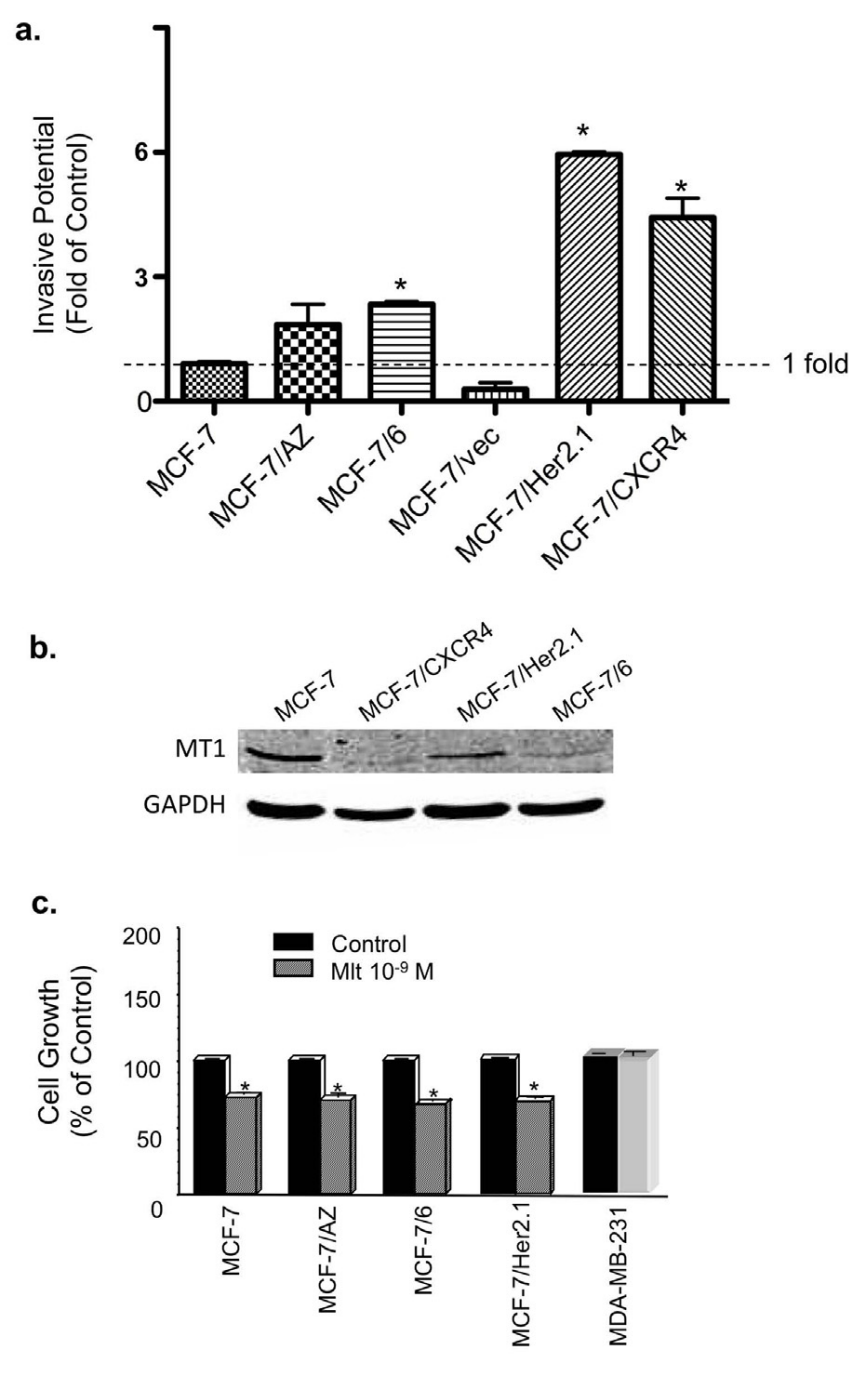
**Melatonin responsiveness and MT1 receptor expression of a panel of human breast cancer cells**. **(a) **The relative invasive potential of MCF-7, MCF-7/AZ, MCF-7/6, MCF-7/vec, MCF-7/Her2.1, and MCF-7/CXCR4 breast cancer cells. Data are presented as fold of control (MCF-7, set as 1). *P < 0.05 versus MCF-7 cells. **(b) **Western blot analysis of MT1 protein expression in MCF-7, MCF-7/CXCR4, MCF-7/Her2.1, and MCF-7/6 cells. Glyceraldehyde-3-phosphate dehydrogenase (GAPDH) protein levels are shown as loading control. **(c) **Effect of melatonin on cell proliferation in MCF-7, MCF-7/AZ, MCF-7/6, MCF-7/Her2.1, and MDA-MB-231 cells. Cells were serum-starved for 24 hours and treated with melatonin (Mlt, 10^-9 ^M) or diluent (Control, 0.00001% ethanol). Cell numbers were determined on day 6 by hemacytometer cell count. Data are presented as percentage of mean cell count in vehicle-treated cells (100%). One-way analysis of variance followed by Student Newman-Keuls *post hoc *test analyses was used to determine statistically significant differences in the cell number between melatonin- and vehicle-treated groups. *P < 0.05 versus vehicle-treated cells (*n *= 3).

Three invasive MCF-7 variants were used in our following studies: the MCF-7/6, MCF-7/Her2.1, and MCF-7/CXCR4 cell lines. Among these three cell lines, MCF-7/Her2.1 is highly invasive as compared with parental MCF-7 cells (sixfold elevated invasion); MCF-7/6 and MCF-7/CXCR4 cells are moderately to highly invasive as compared with MCF-7 cells showing changes in invasive capacity by 2.6- and 4.9-fold, respectively (Figure 1a). Western blot analysis demonstrated that MT1 is expressed in MCF-7, MCF-7/6, MCF-7/Her2.1, and MCF-7/CXCR4 cells, although MCF-7/6 and MCF-7/CXCR4 cells exhibit lower levels of MT1 protein than MCF-7 and MCF-7/Her2.1 cells (Figure 1b). In addition, melatonin treatment after 6 days significantly suppressed cell growth (by 30% to 40%) in all cells examined except the ERα-negative MDA-MB-231 cells, in which no growth inhibition was observed (Figure 1c).

### Phosphorylation of ERK1/2 and p38 MAPKs is upregulated in human breast cancer cells with elevated expression of the Her2/neu or CXCR4

**Figure 2:**
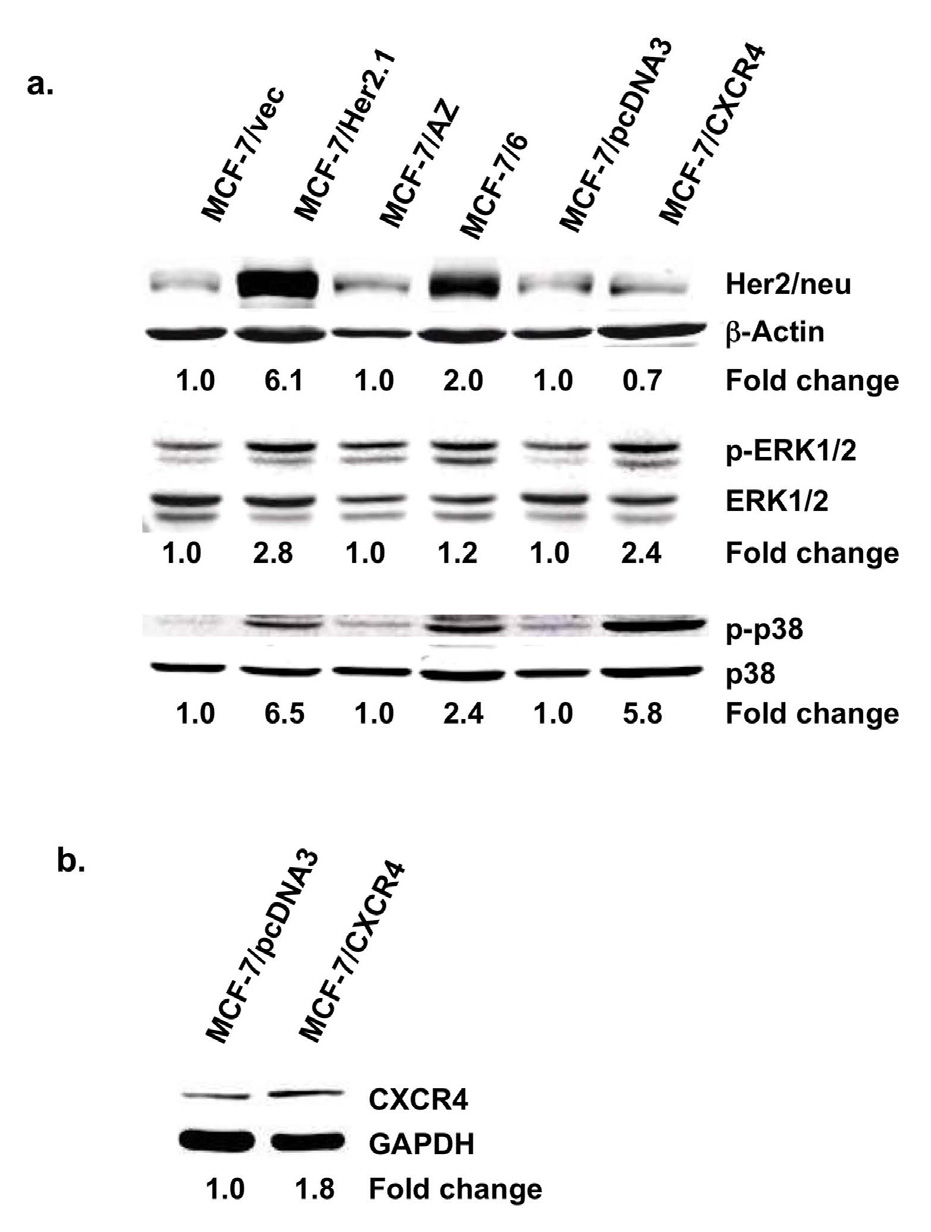
**Elevated phosphorylation of ERK1/2 and p38 MAPK in MCF-7/CXCR4, MCF-7/6, and MCF-7/Her2.1 cells**. **(a) **Western blot analysis of Her2/neu receptor (top), phospho-ERK1/2 (p-ERK1/2) (middle), and phospho-p38 (p-p38) (bottom) in MCF-7/vec, MCF-7/Her2.1, MCF-7/AZ, MCF-7/6, MCF-7/pcDNA3, and MCF-7/CXCR4 breast cancer cells. The band intensity of Her2/neu, phospho-p38, and phospho-ERK1/2 was normalized to that of β-actin, total p38, and ERK1/2, respectively, and expressed as fold of control (MCF-7/vec, MCF-7/AZ, and MCF-7/pcDNA3, set as 1). **(b) **Western blot analysis of CXCR4 expression in MCF-7/pcDNA3 and MCF-7/CXCR4 cells. Levels of glyceraldehyde-3-phosphate dehydrogenase (GAPDH) are shown as loading control. The band intensity of CXCR4 was normalized to that of GAPDH and expressed as fold of control (MCF-7/pcDNA3, set as 1). ERK1/2, extracellular signal-regulated kinase 1/2; MAPK, mitogen-activated protein kinase.

Expression of the Her2/neu and CXCR4 receptor was examined by Western blot analysis. MCF-7/Her2.1 cells showed markedly enhanced expression of Her2/neu as compared with MCF-7/vec cells. Interestingly, Her2/neu expression was also elevated in MCF-7/6 cells as compared with their parental cell line MCF-7/AZ (Figure 2a, top). In addition, CXCR4 expression is elevated by approximately twofold in MCF-7/CXCR4 cells as compared with the MCF-7/pcDNA3 cell line (Figure 2b).

To further investigate whether Her2/neu overexpression leads to upregulation of downstream MAPKs, we examined the phosphorylation of extracellular signal-regulated kinase 1/2 (ERK1/2) and p38 MAPK by Western blot analysis. Phosphorylation of ERK1/2 was elevated in MCF-7/Her2.1, MCF-7/CXCR4, and MCF-7/6 cells by 2.8-, 2.4-, and 1.2-fold, respectively, as compared with their respective control cells (Figure 2a, middle). Moreover, phosphorylation of p38 MAPK (Figure 2a, bottom) was markedly elevated in MCF-7/CXCR4 and MCF-7/Her2.1 cells (5.8- and 6.5-fold, respectively) as compared with their respective control cell lines. The MCF-7/6 cells also exhibited increased p38 phosphorylation (2.4-fold) as compared with MCF-7/AZ cells.

### Melatonin represses breast cancer cell invasion

**Figure 3:**
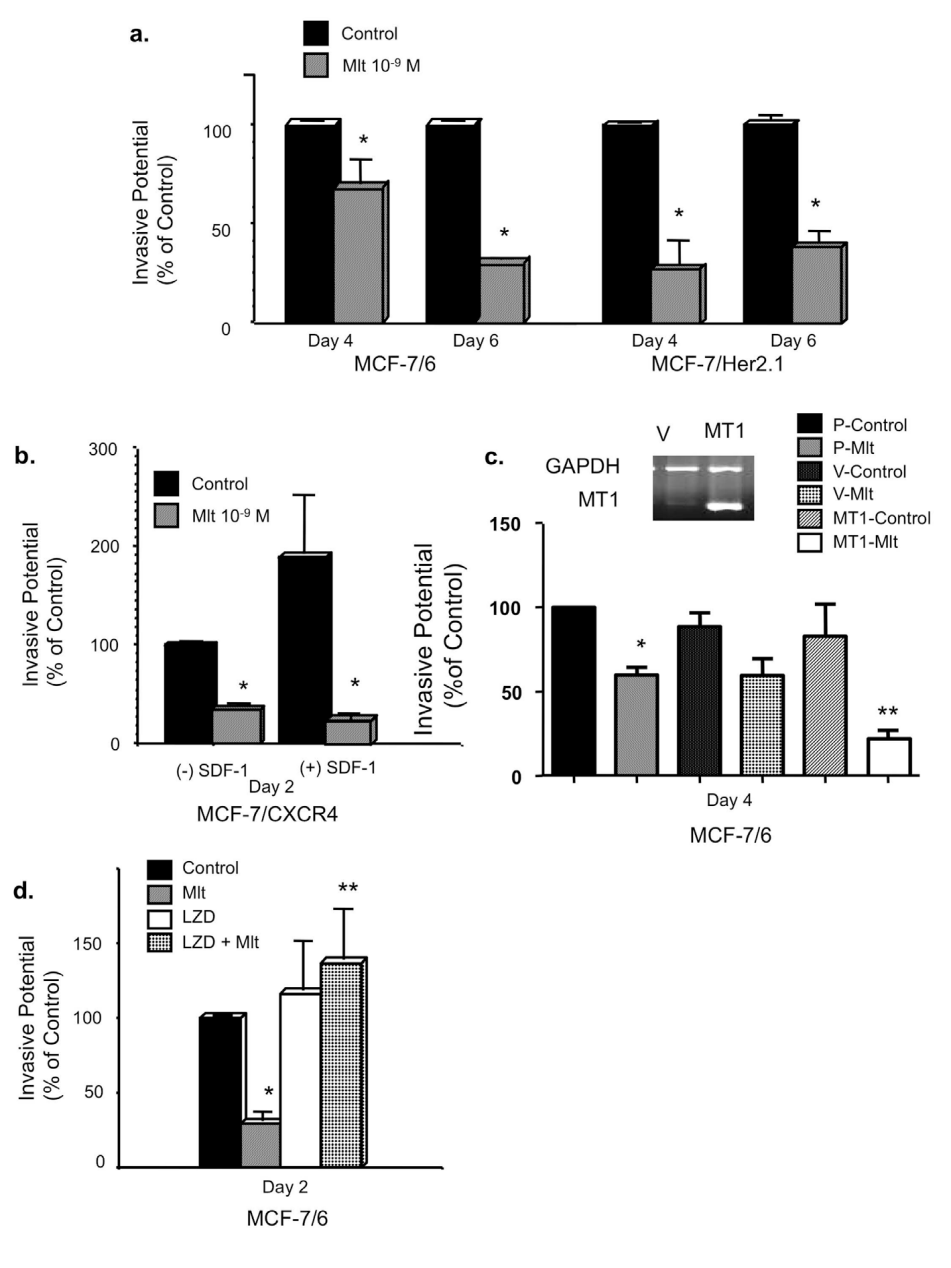
**Melatonin, via MT1, suppresses the invasive potential of human breast cancer cells**. **(a) **Effect of melatonin on the invasion of MCF-7/6 and MCF-7/Her2.1 cells. Cells were plated onto matrigel invasion chambers after being serum-starved for 24 hours and treated with melatonin (Mlt, 10^-9 ^M) or diluent (Control, 0.00001% ethanol) for 4 and 6 days. Data are represented as percentage of control (100%). *P < 0.05 versus diluent-treated cells (*n *= 3). **(b) **Effect of melatonin on stromal-derived factor-1 (SDF-1)-induced invasion of MCF-7/CXCR4 cells. Cells were treated with melatonin (Mlt, 10^-9 ^M) or diluent (Control, 0.00001% ethanol) for 2 days with or without SDF-1 in the bottom chamber as chemoattractant ((+)/(-) SDF-1, 300 ng/mL). Data are presented as percentage of control ((-) SDF-1, vehicle-treated cells, 100%). *P < 0.05 versus diluent-treated cells (*n *= 3). **(c) **Effect of MT1 overexpression on melatonin-regulated invasion of MCF-7/6 cells. Cells were transiently transfected with either pcDNA3.1 empty vector (V) or pcDNA3.1-MT1 plasmid (MT1) or were sham-transfected (P). Twenty-four hours later, cells were plated onto matrigel invasion chambers and treated with melatonin (Mlt, 10^-9 ^M) or diluent (Control, 0.00001% ethanol) for 4 days. Data are presented as percentage of the sham-transfected diluent-treated control (100%). Reverse transcriptase-polymerase chain reaction analysis of MT1 expression is shown to demonstrate the overexpression of MT1 in pcDNA3.1-MT1-transfected cells. *P < 0.05 versus sham-transfected vehicle-treated control cells (P-Control); **P < 0.05 versus vector-transfected melatonin-treated cells (V-Mlt) (*n *= 3). **(d) **Effect of luzindole on melatonin-regulated invasion of MCF-7/6 cells. Cells were treated with diluent (Control, 0.00001% ethanol), melatonin (Mlt, 10^-9 ^M), luzindole (LZD, 10^-8 ^M), or luzindole (10^-8 ^M) for 15 minutes followed by melatonin (10^-9 ^M) treatment (LZD + Mlt) for 2 days. Data are presented as percentage of ethanol-treated control (100%). *P < 0.05 versus vehicle-treated control cells; **P < 0.05 versus melatonin-treated cells (*n *= 3). One-way analysis of variance followed by Student Newman-Keuls *post hoc *test analyses was used to determine statistically significant differences in the percentage of invasive cells among different treatment groups.

Here, we examined the effect of melatonin on the *in vitro *invasion of MCF-7/6, MCF-7/Her2.1, and MCF-7/CXCR4 cells by matrigel invasion chamber assays. Melatonin significantly repressed the invasive potential of all three cell lines. In MCF-7/6 cells, melatonin (10^-9 ^M) significantly suppressed cell invasion by 32% by day 4 and 71% by day 6 (control set as 100%). The same concentration of melatonin inhibited the invasion of MCF-7/Her2.1 cells by 72% by day 4 and 62% by day 6 (Figure 3a). In MCF-7/CXCR4 cells, treatment with 10^-9 ^M melatonin for 2 days significantly decreased cell invasion (by 66%) (Figure 3b).

According to previous studies, invasion of CXCR4-expressing breast cancer cells is enhanced in response to SDF-1/CXCL12 [33]. To investigate whether melatonin inhibits SDF-1-induced invasion of MCF-7/CXCR4 cells, SDF-1 (300 ng/mL) was added to the bottom well of the matrigel invasion chambers as a chemoattractant. SDF-1 increased the invasive potential of MCF-7/CXCR4 cells by 100% as compared with the non-stimulated control (no SDF-1 added to the bottom well and diluent-treated, set as 100%), and this effect was completely blocked by melatonin administration (Figure 3b).

### MT1 receptor mediates melatonin's action on breast cancer cell invasion

We have previously reported that the MT1 but not the MT2 receptor is expressed in MCF-7 cells [32] and that MT1 mediates melatonin's growth-suppressive effect in MCF-7 cells [32,34,35]. We further examined whether the anti-invasive effect of melatonin is also mediated through the MT1 melatonin receptor. As shown in Figure 3c, melatonin treatment induced a significantly enhanced inhibition of cell invasion (73%) in cells that are transiently transfected with and that overexpress MT1 as compared with sham-transfected and vector-transfected cells (approximately 40% inhibition). Moreover, pre-treatment of cells with luzindole, an MT1/MT2 antagonist, for 15 minutes prior to the addition of melatonin completely abrogated melatonin's anti-invasive effect (Figure 3d). These results suggest that the anti-invasive action of melatonin is mediated via the MT1 receptor.

### Melatonin suppresses the expression and activity of MMP-2 and MMP-9

**Figure 4:**
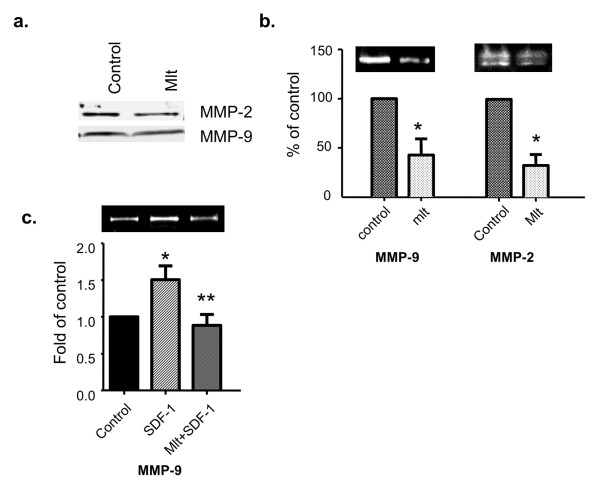
**Melatonin suppresses the expression and activity of MMP-2 and MMP-9 in human breast cancer cells**. Conditioned medium was collected and concentrated as described in Materials and methods. **(a) **Melatonin's effect on the protein expression of MMP-2 and MMP-9. MCF-7/6 cells were treated with melatonin (Mlt, 10^-9 ^M) or diluent (Control, 0.00001% ethanol) for 48 hours. Expression of MMP-2 and MMP-9 in the conditioned medium was analyzed by Western blot analysis using anti-MMP-2 and anti-MMP-9 antibody. **(b) **The effect of melatonin on the activity of MMP-2 and MMP-9. MCF-7/6 cells were treated with melatonin (Mlt, 10^-9 ^M) or diluent (Control, 0.00001% ethanol) for 48 hours. The gelatinase activity of MMP-9 and MMP-2 in the conditioned medium was determined by gelatin zymography. The band intensities of MMP-9 and MMP-2, respectively, are presented in the graph as percentages of control (set as 100%). *P < 0.05 versus diluent-treated control (*n *= 4). **(c) **Effect of melatonin on stromal-derived factor-1 (SDF-1)-induced activity of MMP-9 in MCF-7/CXCR4 cells. MCF-7/CXCR4 cells were treated with diluent (control, 0.00001% ethanol), SDF-1 (100 ng/mL), or melatonin (10^-9 ^M) and SDF-1 (100 ng/mL) simultaneously (Mlt + SDF-1) for 24 hours. The gelatinase activity of MMP-9 in the conditioned medium was determined by gelatin zymography. The band intensity of MMP-9 is presented in the graph as percentage of control (set as 100%). *P < 0.05 versus diluent-treated control; **P < 0.05 versus SDF-1-treated group (*n *= 3). Figures in (a-c) are representative Western blots or gelatin zymograms of at least three independent studies, respectively. MMP, matrix metalloproteinase.

To investigate whether melatonin regulates breast cancer cell invasion by altering the expression of MMPs, particularly MMP-2 and MMP-9, we examined the effect of melatonin on MMP-2 and MMP-9 protein expression in the conditioned medium using MCF-7/6 cells. As determined by Western blot analysis, expression of MMP-9 and MMP-2 was reduced in response to melatonin treatment (10^-9 ^M) (Figure 4a).

We further examined the effect of melatonin on the activity of MMP-2 and MMP-9 by gelatin zymography. As shown in Figure 4b, a significant (60%) decrease in the activity of MMP-9 was observed in cells treated with melatonin (10^-9 ^M) for 48 hours (control set as 100%). Similarly, melatonin also induces a significant decrease in MMP-2 activity (70%) at 48 hours.

Previous studies have shown that one of the mechanisms underlying SDF-1-induced cancer cell invasion is activation of MMP-9 and MMP-2 [33]. Therefore, we further investigated whether melatonin suppresses SDF-1-induced MMP-9 and MMP-2 activity. As determined by gelatin zymography, MMP-9 activity was significantly increased (1.5-fold) after administration of SDF-1 (100 ng/mL) for 24 hours as compared with diluent-treated control (set as 1-fold). The SDF-1-mediated induction of MMP-9 activity was completely blocked by simultaneous treatment with melatonin (Figure 4c). However, melatonin did not block SDF-1-induced MMP-2 activity (data not shown).

### Melatonin's anti-invasive actions are mediated through the p38 MAPK pathway

**Figure 5:**
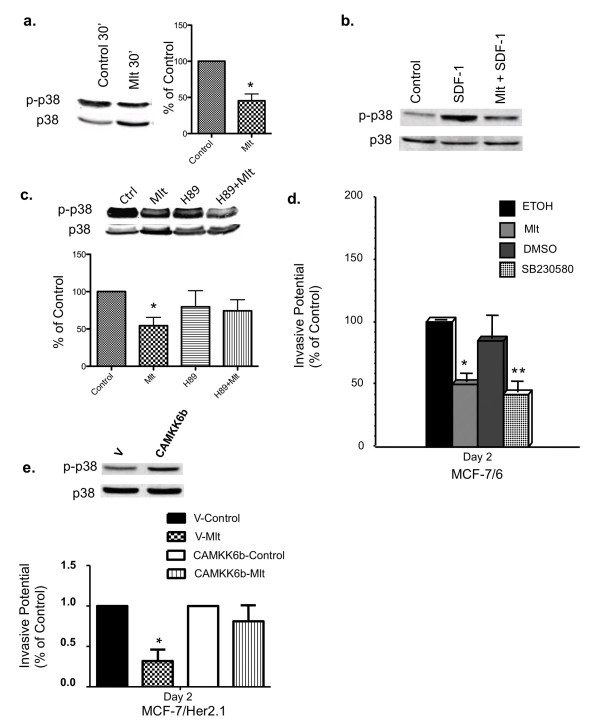
**The anti-invasive effects of melatonin are mediated through the p38 MAPK signaling pathway**. **(a) **The effect of melatonin on the phosphorylation of p38 MAPK. MCF-7/6 cells were serum-starved for 24 hours and treated with diluent (Control, 0.00001% ethanol) or melatonin (Mlt, 10^-9 ^M) for 30 minutes in fresh medium supplemented with 10% fetal bovine serum. Expression of phospho-p38 (p-p38) and total p38 (p38) was analyzed by Western blot analysis. **(b) **The effect of melatonin on stromal-derived factor-1 (SDF-1)-induced p38 phosphorylation. MCF-7/CXCR4 cells were serum-starved for 48 hours and pre-treated with melatonin (10^-9 ^M) or diluent (0.00001% ethanol) for 5 minutes followed by SDF-1 (100 ng/mL) stimulation for 2 minutes (Mlt + SDF-1). **(c) **The effect of H89 on melatonin regulation of p38 phosphorylation. MCF-7/6 cells were serum-starved for 24 hours and pre-treated with H89 (10 μM) for 45 minutes followed by a 30-minute treatment with melatonin (10^-9 ^M). Figures in (a-c) are representative Western blots from three independent studies, respectively. In (a) and (c), the band intensity of phospho-p38 was normalized to that of total p38 and expressed in the graph as percentage of control (set as 100%). *P < 0.05 versus diluent-treated control (*n *= 3). **(d) **The effect of SB230580 on the invasive potential of MCF-7/6 breast cancer cells. MCF-7/6 cells were plated onto matrigel invasion chambers after 24-hour serum starvation and incubated in the medium containing diluent (ETOH, 0.00001% ethanol; dimethyl sulfoxide, or DMSO), melatonin (Mlt, 10^-9 ^M), or SB230580 (20 μM). Data are presented as percentage of ethanol-treated control (100%). *P < 0.05 versus ethanol-treated control cells. **P < 0.05 versus DMSO-treated control cells. **(e) **Effect of CAMKK6b on melatonin's anti-invasive action. MCF-7/Her2.1 cells were transiently transfected with empty vector (V) or CAMKK6b plasmid for 24 hours, plated onto matrigel invasion chambers, and treated with diluent (Control, 0.00001% ethanol) or melatonin (Mlt, 10^-9 ^M) for 2 days. Data are presented as percentage of vector-transfected diluent-treated control (100%). *P < 0.05 versus vehicle-treated control cells. Phosphorylation of p38 MAPK (p-p38) in vector- and CAMKK6b-transfected cells was analyzed by Western blot analysis. Expression of total p38 (p38) was used as loading control. MAPK, mitogen-activated protein kinase.

To investigate whether the MAPKs, ERK1/2 and p38, play a role in melatonin regulation of breast cancer invasion, Western blot analyses were performed to determine whether melatonin regulates the phosphorylation of ERK1/2 and p38 MAPK. Our results demonstrate that melatonin treatment (10^-9 ^M) significantly suppressed p38 phosphorylation in MCF-7/6 cells (Figure 5a) and in MCF-7/Her2.1 cells (data not shown). The melatonin repression of p38 phosphorylation was abrogated by a 45-minute pre-treatment with H89 (10 μM), a protein kinase A (PKA) inhibitor, suggesting that the effect of melatonin on p38 phosphorylation is mediated through PKA (Figure 5c). Furthermore, a robust induction of p38 phosphorylation was observed in MCF-7/CXCR4 cells after 2-minute stimulation with SDF-1 (100 ng/mL), which was blocked by a 5-minute pre-treatment with melatonin (Figure 5b). Conversely, melatonin treatment did not affect the phosphorylation of ERK1/2 in MCF-7/6 cells (data not shown), suggesting that the suppressive effect of melatonin on p38 phosphorylation is specific.

We subsequently conducted matrigel invasion chamber assays to determine whether SB230580, a p38 inhibitor, could mimic melatonin's action on the invasion of MCF-7/6 cells. Treatment of MCF-7/6 cells with melatonin (10^-9 ^M) induced a significant (50%) reduction in the invasive potential of MCF-7/6 cells as compared with diluent-treated controls (ethanol, set as 100%). Similarly, SB230580 significantly inhibited the invasion of MCF-7/6 cells (by 50%) compared with diluent-treated group (dimethyl sulfoxide) (Figure 5d).

The activity of p38 MAPK is regulated by the upstream kinase, MKK6 [36]. To investigate whether upregulation of p38 activity by transfection with a constitutively active MKK6b DNA construct (CAMKK6b) could reverse melatonin's effect on MCF-7/Her2.1 cell invasion, we conducted invasion assays and phosphorylation analyses of p38 by Western blot analyses. As demonstrated in Figure 5e, p38 phosphorylation was upregulated in CAMKK6b-transfected cells. In vector-transfected cells, melatonin treatment significantly suppressed cell invasion (by 70%) as compared with ethanol-treated control (set as 100%). In contrast, melatonin did not have a significant effect on the invasive potential of CAMKK6b-transfected cells (Figure 5e).

### CAMKK6b upregulates MMP-9 mRNA expression and ETS1 phosphorylation

**Figure 6:**
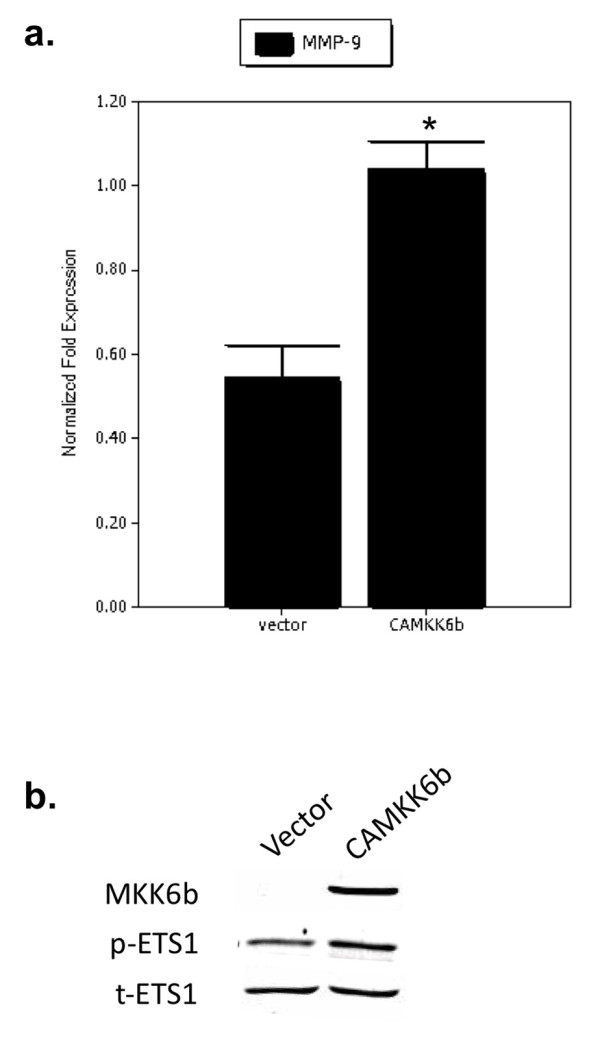
**CAMKK6b upregulates MMP-9 mRNA expression and ETS1 phosphorylation**. **(a) **Effect of CAMKK6b on MMP-9 mRNA expression. MCF-7 cells were transiently transfected with empty vector or CAMKK6b plasmid for 24 hours. Real-time reverse transcriptase-polymerase chain reaction (RT-PCR) analyses were performed to examine the expression of MMP-9 mRNA in vector- and CAMKK6b-transfected MCF-7 cells. The MMP-9 mRNA expression was normalized to levels of 18s ribosomal RNA. *P < 0.05 versus vector-transfected cells. The figure is a representative real-time RT-PCR analysis from three independent studies. **(b) **Effect of CAMKK6b on ETS-1 phosphorylation. MCF-7 cells were transiently transfected with empty vector or CAMKK6b plasmid. Phosphorylation of ETS1 (p-ETS1) in vector- and CAMKK6b-transfected cells was analyzed by Western blot analysis. Expression of total ETS1 (t-ETS1) was used as loading control. The figure is a representative Western blot from three independent studies. MMP, matrix metalloproteinase.

MMP-9 has been shown to be a target gene of the transcription factor ETS1 [37]. There is evidence that p38 can phosphorylate and potentially activate ETS1 [38]. To investigate whether p38 regulates the transcription of MMP-9, real-time RT-PCR analyses were performed to determine the mRNA expression of MMP-9 in MCF-7 cells transiently transfected with CAMKK6b. As shown in Figure 6a, MMP-9 mRNA levels were significantly increased in CAMKK6b-transfected cells as compared with vector-transfected cells. This induction of MMP-9 mRNA expression was accompanied by an increase in the phosphorylation of ETS1 as determined by Western blot analyses (Figure 6b).

## Discussion

To define the role of melatonin in breast cancer cell invasion and metastasis, we used three invasive breast cancer cell clones that were derived from the parental MCF-7 cells - the MCF-7/6, MCF-7/Her2.1, and MCF-7/CXCR4 cells - instead of using the poorly invasive MCF-7 breast cancer cells. These three cell clones were selected because they are highly invasive and metastatic as compared with the parental MCF-7 cells while still retaining many characteristics of the parental MCF-7 cells.

As shown in Figure 1, MT1 is expressed at the protein level in all three cell lines, with MCF-7/Her2.1 cells exhibiting highest level of MT1. In addition, both MCF-7/6 and MCF-7/Her2.1 cells are responsive to melatonin's growth-suppressive effect, with melatonin (10^-9 ^M) showing equal effectiveness in suppressing the growth of these cells as it does in parental MCF-7 cells (Figure 1). When it comes to the ability to invade and metastasize, MCF-7/6, MCF-7/Her2.1, and MCF-7/CXCR4 cells all exhibited elevated invasive potential as compared with the parental MCF-7 cells.

The enhanced invasive potential in MCF-7/Her2.1 and MCF-7/6 cells, respectively, may be a direct result of Her2/neu overexpression. According to previous reports, overexpression of Her2/neu and constitutive activation of the Her2/neu signaling pathway can constitutively activate MAPK [11] while promoting breast cancer cell invasion and metastasis [12-14]. As expected, expression of Her2/neu is markedly upregulated in MCF-7/Her2.1 cells that have been transfected with the wild-type Her2/neu. Interestingly, Her2/neu is also overexpressed in MCF-7/6 cells that have been selected for metastatic potential by serial passaging in nude mice. These data suggest that upregulation of the Her2/neu pathway may be one of the early events in the progression toward invasiveness.

Although the precise mechanisms by which the Her2/neu pathway regulates breast cancer invasion and metastasis are not yet fully understood, it has been suggested that multiple signaling pathways, including ERK1/2 and p38, act as the downstream effectors to promote the invasive potential of these breast cancer cells [15]. Therefore, it is possible that MCF-7/Her2.1 and MCF-7/6 cells acquire their invasive capacities through Her2/neu-induced constitutive activation of ERK1/2 and p38 MAPK. Recent studies have revealed that the chemokine receptor CXCR4 also plays a critical role in the regulation of breast cancer cell invasion and metastasis [16-18]. The molecular mechanisms underlying the action of CXCR4 on breast cancer cell invasion are currently under intense investigation. Given that activation of CXCR4 leads to activation of multiple signaling pathways, including ERK1/2 and p38, in several cell types [19-21], MCF-7/CXCR4 cells may acquire their invasiveness and metastatic potential through CXCR4-mediated upregulation of the ERK1/2 and p38 MAPK signaling pathways.

To define the role of melatonin in breast cancer cell invasion, the effects of melatonin on breast cancer cell invasion were tested on MCF-7/6, MCF-7/Her2.1, and MCF-7/CXCR4 cells by *in vitro *matrigel invasion chamber assays. In agreement with the data reported by Cos and colleagues [3], we have demonstrated that melatonin, at physiological concentrations (10^-9 ^M), significantly inhibits the invasion of MCF-7/6, MCF-7/Her2.1, and MCF-7/CXCR4 breast cancer cells. Furthermore, melatonin blocked the SDF-1/CXCL12-induced invasive potential of MCF-7/CXCR4 cells. Melatonin's anti-invasive action is also reflected by its suppressive effects on the expression of MMP-2 and MMP-9, two major MMPs mediating the degradation of the extracellular matrix. Several reports have indicated that melatonin regulates the activity of MMP-9 during protection against ethanol-induced gastric ulcer and endometriosis [39,40]. In our study, melatonin treatment not only reduces the protein expression but also represses the enzymatic activity of MMP-2 and MMP-9. These results suggest that melatonin's anti-invasive action is mediated, at least in part, by diminishing the ability of breast cancer cells to degrade the components of extracellular matrix by modulating MMP-2 and MMP-9 expression and activity. Moreover, previous studies have shown that the activities of MMPs are regulated by a group of endogenous molecules, namely, the tissue inhibitors of matrix metalloproteinase (TIMPs) [41]. According to our unpublished data from cDNA microarray analysis, TIMP-3 expression is upregulated in melatonin-treated MCF-7 cells overexpressing the MT1 receptor, suggesting that TIMP-3 may be another target of melatonin's anti-invasive action.

To delineate the signaling pathway(s) used by melatonin to affect the invasive capacity of breast cancer cells, we first investigated whether melatonin's anti-invasive action is an MT1 receptor-mediated event. We have previously reported that the MT1 but not the MT2 receptor is expressed in MCF-7 cells [32] and that MT1 mediates melatonin's growth-suppressive effect in MCF-7 cells [32,34,35]. It appears that MT1 is also involved melatonin's anti-invasive actions. As demonstrated in our studies, enhanced MT1 expression potentiated melatonin-mediated inhibition of cell invasion in MCF-7/6 cells. In contrast, this inhibitory effect was abolished by pre-treatment of the cells with luzindole, an MT1/MT2 antagonist. The above data suggest that melatonin's anti-invasive action is mediated, at least in part, through the G-protein-coupled MT1 receptor.

A well-established key intracellular signaling pathway downstream of the MT1 receptor is the cAMP/PKA pathway. As we previously reported, the MT1 receptor, by coupling to G_i _proteins upon melatonin binding, blocks the accumulation of cAMP and potentially inhibits the activity of PKA in MCF-7 cell [31]. Considering that the cAMP/PKA pathway cross-talks with diverse signaling pathways, including the ERK1/2 and p38 MAPK, and drawing inspiration from our observation that constitutive activation of ERK1/2 and p38 MAPK appears to be the driving force to promote the invasion of MCF-7/6, MCF-7/CXCR4, and MCF-7/Her2.1 cells, we hypothesized that melatonin inhibits the invasion of these cells by interacting with the ERK1/2 and p38 MAPK pathways.

Melatonin regulation of MAPKs has been observed in several cell types, including the COS-7 cell and brain tissue, where melatonin has been reported to increase the phosphorylation of ERK1/2 and JNK (c-Jun N-terminus kinase) [42,43]. In human adult mesenchymal stem cells, the phosphorylation of MEK1/2 and ERK1/2 is increased by acute melatonin exposure but is inhibited by the long-term melatonin administration [44]. However, our studies show that the anti-invasive action of melatonin is mediated through the p38 MAPK pathway and not the ERK1/2 pathway since melatonin treatment (10^-9 ^M) repressed p38 phosphorylation in both MCF-7/6 (Figure 5a) and MCF-7/Her2.1 (data not shown) breast cancer cells but had no effect on ERK1/2 phosphorylation (data not shown). Furthermore, the p38 inhibitor, SB230580, mimicked melatonin's effect on cell invasion, significantly reducing the invasive potential of the MCF-7/6 cells (Figure 5d), while expression of the constitutively active MKK6b blocked melatonin's effect on MCF-7/Her2.1 cell invasion (Figure 5e).

Contrary to what we had expected, expression of CAMKK6b did not significantly induce the invasive potential of MCF-7/Her2.1 cells. This may be due to the hyper-activation of p38 MAPK in these Her2/neu-overexpressing cells (6.5-fold elevated phosphorylation compared with control cell line), which has reached a maximum for its stimulatory effect on cell invasion such that further elevation of p38 activity beyond the maximum does not necessarily lead to further increase in the invasive potential of these already highly invasive breast cancer cells.

Several lines of evidence suggest that p38 MAPK regulates the expression of MMP-9 [25,45]. We have shown that expression of CAMKK6b leads to increased MMP-9 mRNA expression and elevated ETS1 phosphorylation in MCF-7 cells (Figure 6). Given that MMP-9 is an ETS1 target gene [37], these results suggest that the MKK6/p38 pathway may regulate MMP-9 transcription through phosphorylation and potentially activation of ETS1.

In summary, the above data suggest that melatonin, via its MT1 receptor, plays an inhibitory role in breast cancer cell invasion, possibly by specifically downregulating the p38 MAPK signaling pathway. Although the precise mechanism or mechanisms underlying melatonin's action on p38 MAPK remain unknown, one possibility is that melatonin regulation of p38 phosphorylation is mediated through G_i _protein-induced changes in cAMP level and PKA activity since melatonin's effect on p38 phosphorylation is attenuated by PKA inhibitor H89 (Figure 5c).

**Figure 7:**
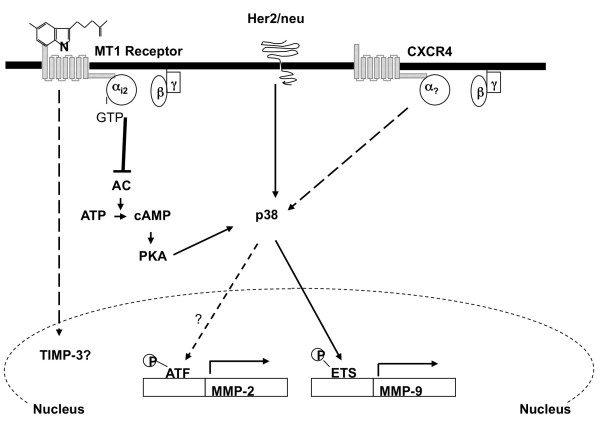
**Model of melatonin regulation of breast cancer cell invasion**. Ligand-dependent activation of the membrane-associated G-protein-coupled receptor MT1 leads to coupling of G_i2 _protein to MT1 receptor. As a result, the Gα_i2 _subunit dissociates from the Gβγ subunits and inhibits the activity of adenylcyclase (AC), and this leads to a decrease in the intracellular level of cAMP and inhibition of protein kinase A (PKA) activity. The cAMP/PKA pathway cross-talks with the p38 pathway through PKA. In response to the reduced cAMP level, activity of p38 is suppressed, and this causes further downregulation of MMP-9 expression via repression of ETS1 transcriptional activity and, potentially, downregulation of MMP-2 transcription. MMP, matrix metalloproteinase.

In recent years, the p38 MAPK has emerged as a key signaling molecule in the regulation of cancer invasion and metastasis by modulating the expression and activity of molecules governing the degradation of extracellular matrix (that is, urokinase plasminogen activator, MMP-2, and MMP-9) [22-25,45]. Our studies show that phosphorylation of p38 MAPK, but not ERK1/2, is dramatically elevated in both Her2/neu-overexpressing (MCF7/6 and MCF-7/Her2.1) and CXCR4-overexpressing (MCF-7/CXCR4) cells. These data indicate that, although MCF-7/6, MCF-7/Her2.1, and MCF-7/CXCR4 cells acquire their malignant phenotypes through different approaches, constitutive activation of p38 MAPK may be a common mechanism driving the transition of these cells from the poorly/non-invasive phenotype to an invasive phenotype (Figure 7). By inhibiting p38 phosphorylation, melatonin has emerged as a promising anti-invasion factor that may be useful in future cancer therapeutics in prevention not only of breast cancer [46] but also of the 'non-invasive-to-invasive' transition. In addition, cancer metastasis is a multi-step event. Although our studies have clearly demonstrated that melatonin significantly inhibits breast cancer cell invasion *in vitro*, it is critical to further define the role of melatonin in regulating breast cancer metastasis *in vivo *to evaluate the clinical significance of melatonin's anti-invasive effect.

## Conclusions

In the present studies, we investigated the role of melatonin in the regulation of breast cancer cell invasion. Our results demonstrated that melatonin, via its MT1 receptor, plays an inhibitory role in breast cancer cell invasion, possibly by specifically downregulating the p38 MAPK signaling pathway, and the downstream activity of MMP-2 and MMP-9.

## Abbreviations

CT: threshold cycle; DMEM: Dulbecco's modified eagle medium; ERα: estrogen receptor-alpha; ERK1/2: extracellular signal-regulated kinase 1/2; FBS: fetal bovine serum; MAPK: mitogen-activated protein kinase; MMP: matrix metalloproteinase; PCR: polymerase chain reaction; PKA: protein kinase A; RT-PCR: reverse transcriptase-polymerase chain reaction; SDF-1: stromal-derived factor-1; TIMP: tissue inhibitor of matrix metalloproteinase.

## Competing interests

The authors declare that they have no competing interests.

## Authors' contributions

LM participated in the overall study design, carried out the molecular studies, and drafted the manuscript. LY carried out the RT-PCR analysis. LMS performed some of the Western blot analyses. FEJ and MEB critically revised the manuscript. SMH conceived the study and participated in its design and coordination and helped to draft the manuscript. All authors read and approved the final manuscript.
